# Granulation Patterns of Functional Corticotroph Tumors Correlate with Tumor Size, Proliferative Activity, T2 Intensity-to-White Matter Ratio, and Postsurgical Early Biochemical Remission

**DOI:** 10.1007/s12022-024-09819-y

**Published:** 2024-07-24

**Authors:** Elif Tutku Durmuş, Mehmet Kefeli, Ozgur Mete, Sultan Çalışkan, Kerim Aslan, Mustafa Arda Onar, Ramis Çolak, Buğra Durmuş, Cengiz Cokluk, Ayşegül Atmaca

**Affiliations:** 1 Department of Endocrinology and Metabolism, Faculty of Medicine, Ondokuz Mayis University, 55200 Atakum Samsun, Turkey; 2 Department of Pathology, Faculty of Medicine, Ondokuz Mayis University, Samsun, Turkey; 3 Department of Laboratory Medicine and Pathobiology, University of Toronto, Toronto, ON Canada; 4 Department of Pathology, University Health Network, 200 Elizabeth Street, 11 Floor, Toronto, ON M5G 2C4 Canada; 5 Endocrine Oncology Site Group, Princess Margaret Cancer Centre, Toronto, ON Canada; 6 Department of Radiology, Faculty of Medicine, Ondokuz Mayis University, Samsun, Turkey; 7 Department of Neurosurgery, Faculty of Medicine, Ondokuz Mayis University, Samsun, Turkey

**Keywords:** Cushing disease, Densely granulated corticotroph tumor, Sparsely granulated corticotroph tumor, Early biochemical remission, Tumor size, Proliferative activity, TPIT, T2 intensity, Corticotroph adenoma, Pituitary adenoma, Pituitary tumor, Pituitary neuroendocrine tumor, Corticotroph tumor

## Abstract

Unlike somatotroph tumors, the data on correlates of tumor granulation patterns in functional TPIT lineage pituitary neuroendocrine tumors (corticotroph tumors) have been less uniformly documented in most clinical series. This study evaluated characteristics of 41 well-characterized functional corticotroph tumors consisting of 28 densely granulated corticotroph tumors (DGCTs) and 13 sparsely granulated corticotroph tumors (SGCTs) with respect to preoperative clinical and radiological findings, tumor proliferative activity (including mitotic count and Ki-67 labeling index), and postoperative early biochemical remission rates. The median (interquartile range (IQR)) tumor size was significantly larger in the SGCT group [16.00 (16.00) mm in SGCT vs 8.5 (9.75) mm in DGCT, *p* = 0.049]. T2-weighted signal intensity and T2 intensity (quantitative) did not yield statistical significance based on tumor granulation; however, the T2 intensity-to-white matter ratio was significantly higher in SGCTs (*p* = 0.049). The median (IQR) Ki-67 labeling index was 2.00% (IQR 1.00%) in the DGCT group and 4.00% (IQR 7.00%) in the SGCT group (*p* = 0.043). The mitotic count per 2 mm^2^ was higher in the SGCT group (*p* = 0.001). In the multivariate analysis, the sparse granulation pattern (SGCT) remained an independent predictor of a lower probability of early biochemical remission irrespective of the tumor size and proliferative activity (*p* = 0.012). The current study further supports the impact of tumor granulation pattern as a biologic variable and warrants the detailed histological subtyping of functional corticotroph tumors as indicated in the WHO classification of pituitary neuroendocrine tumors. More importantly, the assessment of the quantitative T2 intensity-to-white matter ratio may serve as a preoperative radiological harbinger of SGCTs.

## Introduction

Functional corticotroph tumors are pituitary neuroendocrine tumors (PitNETs) of the TPIT lineage that cause Cushing’s disease (CD) [1]. The distinction of the pituitary origin of hypercortisolism and the histopathological identification of a corticotroph tumor are important steps in clinical management [2]. At the histopathology level, corticotroph tumors are subtyped based on the distribution and extent of adrenocorticotrophic hormone (ACTH)-containing granules as densely granulated corticotroph tumors (DGCTs), sparsely granulated corticotroph tumors (SGCTs), and Crooke’s cell tumors. Traditionally, DGCTs have been linked to florid Cushing’s disease and smaller tumors, while SGCTs can be associated with attenuated Cushing’s symptoms and larger tumors [3, 4]. Crooke’s cell tumors, which are considered rare and biologically aggressive tumor subtypes, tend to be more frequently associated with cyclic Cushing’s symptoms [5]. Unlike somatotroph tumors, the extent of the data on clinicopathological correlates of granulation patterns of corticotroph tumors is less uniformly documented in most clinical series. Earlier studies showed some correlations with respect to the extent and size of secretory/prosecretory granules, tumor invasiveness, and proliferative activity of corticotroph tumors [6, 7]. More recent studies conducted in light of the detailed histological subtyping of these tumors further supported clinicopathological correlations between tumor granulation patterns and tumor size as well as tumor invasiveness [8–10]. In addition, enrichment of some molecular alterations (e.g., *USP8*) particularly in small (< 1 cm) corticotroph tumors is also a focus of interest [4, 11].

The extent of tumor invasion and resectability (being worse in tumors invading cavernous sinus and surroundings) are well-established parameters interfering with the structural recurrence of PitNETs. The establishment of preoperative harbingers of tumor granulation patterns of corticotroph tumors and additional predictors of postoperative biochemical remission can guide further management of CD by influencing the follow-up process, facilitating individualized treatment strategies, and providing realistic expectations for the patient and managing physicians [1, 12–14]. In some series, radiological characteristics particularly tumor size and tumor invasiveness, preoperative ACTH and postoperative immediate morning cortisol levels, and absence of Crooke’s hyaline change of the non-tumorous corticotrophs were shown to correlate with the status of postoperative remission in CD [14–19]. By taking into account the common challenges in the clinical management of patients with CD, this study investigated whether the tumor granulation patterns show any predictive correlation with demographic data, hormonal values, magnetic resonance imaging (MRI) findings, tumor proliferative activity, and postoperative early biochemical remission in a series of 41 well-characterized corticotroph tumors.

## Materials and Methods

### Patient Characteristics and Inclusion Criteria

Upon institutional research ethics approval, a retrospective review of the pathology files identified 72 corticotroph tumors (out of approximately 550 transsphenoidal pituitary tumor resection specimens) diagnosed between 2008 and 2019 at the Ondokuz Mayis University, Department of Pathology. Patients with Crooke’s cell tumors and silent corticotroph tumors, those with postoperative follow-up time of < 6 months, and those with missing/incomplete clinical, radiological, or pathology data were excluded from the study. As a consequence, a series of 41 adult CD patients (> 18 years) consisting of 28 DGCTs and 13 SGCTs were included. Several clinical, biochemical, radiological, and pathological variables collected in this series are summarized below.

### Clinical and Biochemical Variables

Patients with CD were diagnosed according to static and dynamic biochemical laboratory results, pituitary MRI findings, and relevant clinical signs and symptoms. Standard biochemical screening was performed according to the guidelines [20], including increased excretion of 24-h urinary free cortisol (UFC) (at least two measurements), abnormal circadian rhythm with late-night salivary cortisol (at least two measurements), and impaired glucocorticoid feedback with an overnight dexamethasone suppression test (ODST; 1 mg at midnight) and/or low-dose dexamethasone suppression test (LDDST; 2 mg/day for 48 h). Further examinations were performed for patients with concordant positive results from at least two different tests (increased serum ACTH levels, serum cortisol, or UFC suppression of > 50% with a high-dose dexamethasone suppression test (8 mg/day for 48 h), increased plasma ACTH, and increased cortisol concentrations after corticotropin-releasing hormone administration). Bilateral inferior petrosal sinus sampling was performed in cases of normal pituitary MRI results or intrasellar tumors of ≤ 6 mm in patients biochemically diagnosed with CD [1]. The recognition of early biochemical remission was based on hormonal assessments performed 6 months after the first pituitary surgery for CD, with the definition being based on clinical and biochemical evidence of adrenal insufficiency. For patients with preservation of adrenal function, early biochemical remission was recognized in the event of biochemical evidence of eucortisolemia (24-h UFC, morning serum cortisol and plasma ACTH levels within their respective reference ranges, and preserved circadian rhythm of serum cortisol and serum cortisol levels suppressed to ≤ 1.8 µg/dL with the ODST) [9, 10]. Persistent CD was defined as no clinical or biochemical remission after the first pituitary surgery; recurrence was defined as the reappearance of clinical and biochemical features of hypercortisolism after a period of remission after the first pituitary surgery [1, 13, 16, 21]. For patients with recurrence or persistent disease, a minimum of one treatment option was applied during follow-up, including a second transsphenoidal surgery (TSS), bilateral adrenalectomy, medical treatment options, or radiotherapy [12].

### Magnetic Resonance Imaging (MRI) Studies

A 3-T Ingenia scanner (Philips Healthcare) with a 32-channel head coil was used to conduct the MRI examinations. One neuroradiologist and one general radiologist independently and randomly reviewed the pituitary MRI scans. These investigators were blinded to the clinicopathological and laboratory findings. T2W sequences were used in the qualitative examination of pituitary tumors, which were classified according to the categorization previously suggested by Potorac et al. [22] as hypointense, isointense, or hyperintense in comparison to normal pituitary glands. Independent radiologists conducted the quantitative evaluations, which included measurements of the tumor’s T2W signal intensities. Circular regions of interest (ROIs) were manually established solid portions, excluding the necrotic, cystic, and calcified portions of the tumors from the T2W sequences. Both T2W and contrast-enhanced T1W images were used for the identification of these areas. ROIs were manually established, and T2W signal intensities were evaluated for temporal white matter, allowing the subsequent calculation of the ratio of the tumor’s signal intensity to that of the temporal white matter [23]. We applied the Knosp scale to estimate invasive growth. The Knosp grading system facilitates evaluations of tumor invasion into the cavernous sinus, and tumors of grades III and IV are accepted as invasive growths [24, 25]. In the evaluation of tumor invasion into the sphenoid bone, the Hardy grading system was applied, with tumors of grades III and IV accepted as invasive. Grades A, B, and C reflect suprasellar extension according to the Hardy system, whereas the Hardy grades D and E are indicative of parasellar extension [25, 26]. Manual segmentation of the pituitary was carried out using ITK-SNAP Version 3.8.0-beta software [27]; the targeted anatomical region was outlined, and then, the volume of the delineated region was automatically calculated in cubic millimeters.

### Pathological Variables

**Fig. 1:**
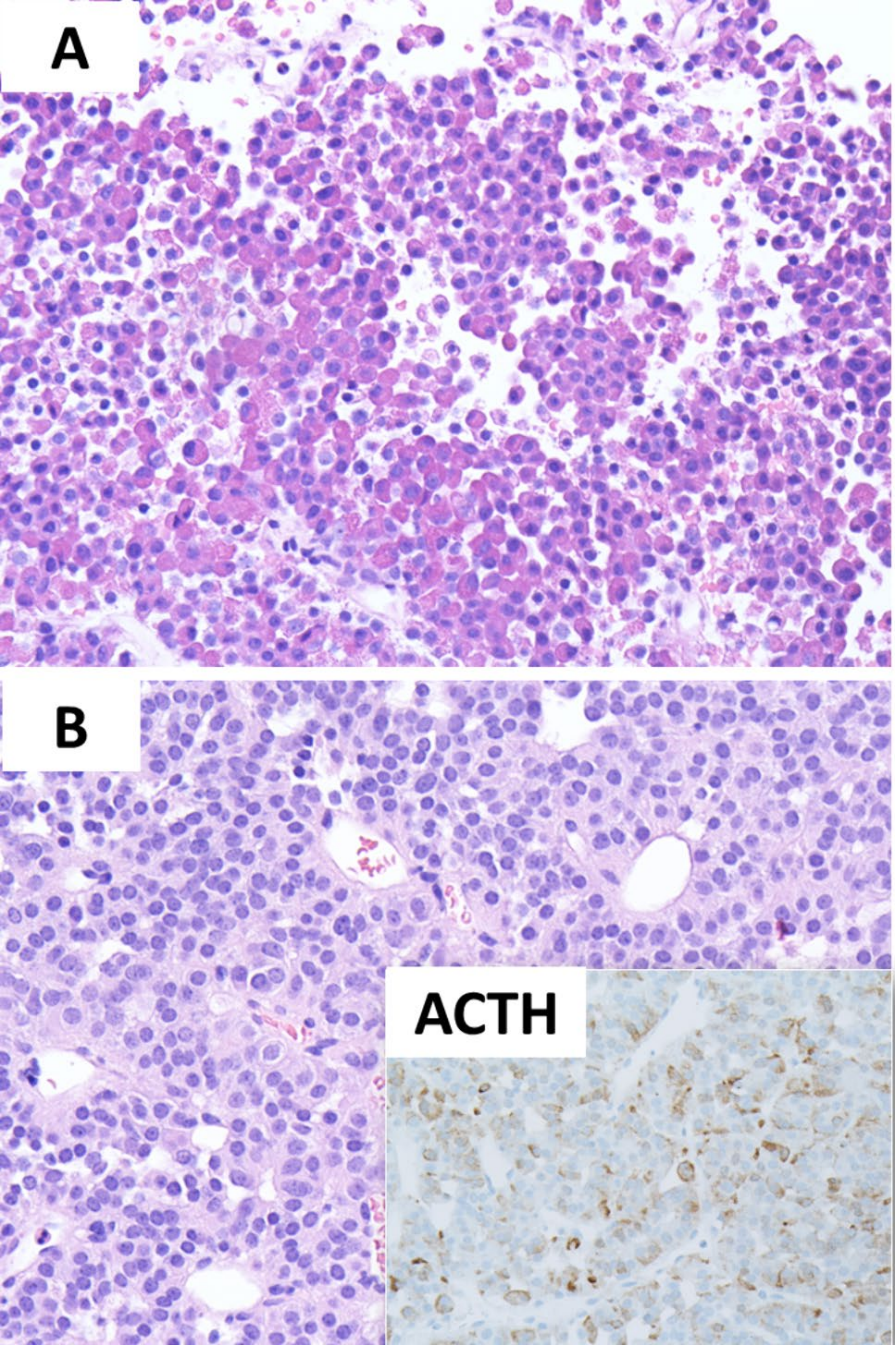
Histopathological characteristics of corticotroph tumors. This composite figure illustrates features of densely granulated (**A**) and sparsely granulated (**B**) corticotroph tumors. Densely granulated corticotroph tumors are enriched in basophilic granules that can be easily identifiable on H&E-stained sections (**A**) and are diffusely positive for PAS (not shown herein) and ACTH (not shown herein). Sparsely granulated corticotroph tumors have a lightly basophilic granular cytoplasm reflecting their sparse granulation pattern which can be better visualized on ACTH immunohistochemistry (inset on **B**)

Pathological variables (tumor subtype and tumor proliferation) were recorded for 41 pituitary tumors. The diagnosis of corticotroph tumor was confirmed using morphological findings as well as histochemical (PAS and reticulin) and immunohistochemical biomarkers including adenohypophyseal transcription factors and hormones as well as Ki-67 and CAM5.2 [3]. Given the retrospective nature of the data collection, the specific details of antibodies used varied due to changes in suppliers, but all biomarkers underwent rigid validation to be used in a clinical diagnostic immunohistochemistry laboratory. All tumors were subtyped either as DGCT or SGCT based on the extent of ACTH and PAS-positive cytoplasmic granules [3, 28]. DGCTs were composed of basophilic tumor cells (Fig. 1A) showing strong/diffuse to near diffuse PAS and ACTH reactivity, whereas SGCTs consisted of lightly basophilic cells with variable, often focal/weak PAS and ACTH reactivity (Fig. 1B). Negativity for GATA3, PIT1, and SF1 and adenohypophyseal hormones other than ACTH ensured the lack of PitNETs with multilineage differentiation or multiple PitNETs of distinct lineages [29, 30]. Tumor proliferation was assessed using both conventional mitotic count (mitotic count per 2 mm^2^, based on mean mitotic count per 10 mm^2^ from areas of high mitotic density) and Ki-67 (manual counting in 1000 tumor cells from hot spots). The Ki-67 labeling index (LI) was graded in two categories as < 3% or ≥ 3% positive nuclei, while the mitotic count was categorized as < 2 or ≥ 2 mitoses per 2 mm^2^. The Ki-67 LI and mitotic count median values and subcategories were compared between the groups.

### Data Analyses

Histological tumor subtypes (DGCT vs SGCT) were compared using preoperative clinical and biochemical findings, proliferative activity, radiological findings (tumor size, tumor invasion, and tumor intensity on MRI), and the status of postoperative early biochemical remission. IBM SPSS Statistics 25.0 for Windows (IBM Corp.) was utilized for all statistical analyses. In the presentation of descriptive statistics, categorical variables were given as numbers and percentages, while continuous variables were given as mean ± standard deviation and median (interquartile range (IQR)). Continuous variables were analytically evaluated for the presence of normal distribution using the Shapiro–Wilk tests and the Kolmogorov–Smirnov tests. Independent *t*-tests were conducted for comparative analyses of two independent groups of data with normal distribution, and the Mann–Whitney *U* tests were performed for comparative analyses of two independent groups of data with non-normal distribution. For categorical variables, chi-squared tests were performed for comparative analyses of independent groups. For categorical comparisons, *p*-values determined by Fisher’s exact test are shown in Table 1. Variables confirmed to be statistically significant (*p* < 0.05) by univariate analysis were subsequently incorporated into a multivariate logistic regression model to determine the variables that were predictive of the tumor subtypes.

## Results

### Demographical and Follow-Up Data

The mean age at the time of histological diagnosis was 40.73 ± 14.57 years. This cohort showed a female predominance (85.4%, *n* = 35). The mean age for female patients was 42.54 ± 14.40 years, whereas the mean age for males was 30.16 ± 11.44 years. Before the scheduled pituitary surgery, 2 patients with macrotumors (> 1.0 cm tumors) manifested with pituitary apoplexy. The mean follow-up period was 69.14 ± 31.50 months. Postoperative early biochemical remission was detected in 26 (63.4%) cases, persistent disease was observed in 15 (36.6%) cases, and recurrence developed in 5 (12.2%) cases during the follow-up. Reoperation was performed during the care of 15 patients. Additional medical treatment (single or combined pasireotide, cabergoline, ketoconazole, or metyrapone) was prescribed for 9 patients, radiotherapy was administered for 5 patients, and bilateral adrenalectomy was performed for 2 patients.

### Variables with Respect to Tumor Subtypes

All clinical, radiological, and histopathological features are shown in Table 1.

**Table 1:** Comparison of clinical, radiological, and histopathological characteristics with respect to tumor subtypes

	**Total (*n* = 41)**	**DGCTs (*n* = 28)**	**SGCTs (*n* = 13)**	***p*-value**
**Clinical characteristics**				
Age at diagnosis (years) (mean ± SD)	40.73 ± 14.57	39.50 ± 15.18	43.38 ± 13.34	0.434^b^
Gender, Female (*n*%)	35 (85.4)	23 (65.7)	12 (34.3)	0.645^a^
Serum cortisol (6.2–19.4 µg/dl) (mean ± SD)	22.23 ± 8.89	21.97 ± 9.49	22.80 ± 7.75	0.783^b^
Serum ACTH (0–46 pg/ml) median (IQR)	71.30 (56.70)	58.80 (51.18)	78.50 (62.70)	0.377^c^
24-h UFC (10–100 µg/dl) (mean ± SD)	369.96 ± 313.77	407.95 ± 334.93	243.33 ± 233.77	0.450^b^
LDDST (µg/dl) median (IQR)	10.50 (12.75)	11.13 (15.60)	14.00 (11.00)	0.937^c^
Outcomes (*n*%)				
Remission	26 (63.4)	23 (88.5)	3 (11.5)	** < 0.001**^**d**^
Persistence disease	15 (36.6)	5 (33.3)	10 (66.7)	
**Radiological characteristics**				
Size of tumor *n*(%)				
Microtumor	18 (50)	14 (77.8)	4 (22.2)	0.278^d^
Macrotumor	18 (50)	11 (61.1)	7 (38.9)	
Largest tumor size (mm) median (IQR)	10.00 (12.00)	8.50 (9.75)	16.00 (16.00)	**0.049**^**c**^
Tumor volume (mm^3^) median (IQR)	286.00 (1840.00)	281.00 (898.25)	670.00 (3436.50)	0.207^c^
T2-weighted signal intensity *n*(%)				
Hypointense	13 (36.1)	9 (69.2)	4 (30.8)	0.903^a^
Isointense	12 (33.3)	9 (75.0)	3 (25.0)	
Hyperintense	11 (30.6)	7 (63.6)	4 (36.4)	
Invisible tumor (*n*%)	5 (12.2)	3 (60.0)	2 (40.0)	0.645^a^
T2 intensity (quantitative) (mean ± SD)	637.83 ± 340.72	667.33 ± 296.01	579.45 ± 410.31	0.560^b^
White matter (quantitative) (mean ± SD)	439.05 ± 215.90	561.83 ± 218.47	359.09 ± 180.30	**0.025**^**b**^
T2 intensity/white matter (mean ± SD)	0.04 ± 0.02	0.03 ± 0.02	0.05 ± 0.21	**0.049**^**b**^
Sphenoid sinus invasion *n*(%)	4 (17.4)	2 (50.0)	2 (50.0)	1.000^a^
Knosp classification grades (*n*%)				1.000^a^
Non-invasive (0–1-2)	17 (73.9)	9 (52.9)	8 (47.1)	
Invasive (3–4)	6 (26.1)	3 (50.0)	3 (50.0)	
Hardy classification grades *n*(%)				1.000^a^
Non-invasive (0–1-2)	21 (91.3)	11 (52.4)	10 (47.6)	
Invasive (3–4)	2 (8.7)	1 (50.0)	1 (50.0)	
Hardy classification stages *n*(%)				0.478^a^
Suprasellar (A-B-C)	22 (95.6)	12 (54.5)	10 (45.5)	
Parasellar (D-E)	1 (4.4)	0 (0.0)	1 (100.0)	
**Other histopathological characteristics**				
Ki-67 labeling index median (IQR)	2.00 (3.00)	2.00 (1.00)	4.00 (7.00)	**0.043**^**c**^
< 3% *n*(%)	22 (53.7)	17 (77.3)	5 (22.7)	0.184^d^
≥ 3%	19 (46.3)	11 (57.9)	8 (42.1)	
Mitotic count (per 2mm^2^) median (IQR)	0.00 (1.00)	0.00 (1.00)	1.00 (2.50)	**0.001**^**c**^
< 2 *n*(%)	34 (82.9)	27 (79.4)	7 (20.6)	**0.002**^**a**^
≥ 2	7 (17.1)	1 (14.3)	6 (85.7)	

Bold values denote statistical significance

*24-h UFC* 24-h urinary free cortisol, *ACTH* adrenocorticotropic hormone, *DGCTs* densely granulated corticotroph tumors, *LDDST* low-dose dexamethasone suppression test, *SGCTs* sparsely granulated corticotroph tumors

^a^Fisher’s exact test

^b^Independent *t*-test

^c^Mann Whitney *U* test

^d^Pearson’s chi-squared test

### Epidemiology and Laboratory Findings

Twenty-eight (68.3%) patients had DGCTs, and the remaining 13 had SGCTs (31.7%). The mean age of the DGCT group was 39.50 ± 15.18 years, while it was 43.38 ± 13.34 years in the SGCT group. Although the age at diagnosis was slightly higher in the SGCT group, the difference was not significant (*p* = 0.434). While the median (IQR) ACTH value was 58.80 (51.18) pg/mL in the DGCT group, it was 78.50 (62.70) pg/mL in the SGCT group, but this did not yield any significance. Gender distribution and serum cortisol, 24-h UFC, and LDDST values were similar between both tumor groups (*p* > 0.05). Early biochemical remission after surgery was detected in 23 cases (88.5%) in the DGCT group, while it was detected in only 3 (11.5%) SGCTs (*p* < 0.001) (Table 1). Four (80%) of the five patients with recurrent disease identified during the follow-up period had DGCTs. The 2 patients with DGCTs, which manifested with pituitary apoplexy, had macrotumors in imaging studies. Two patients who died during follow-up time had SGCTs.

### Imaging Findings

**Fig. 2:**
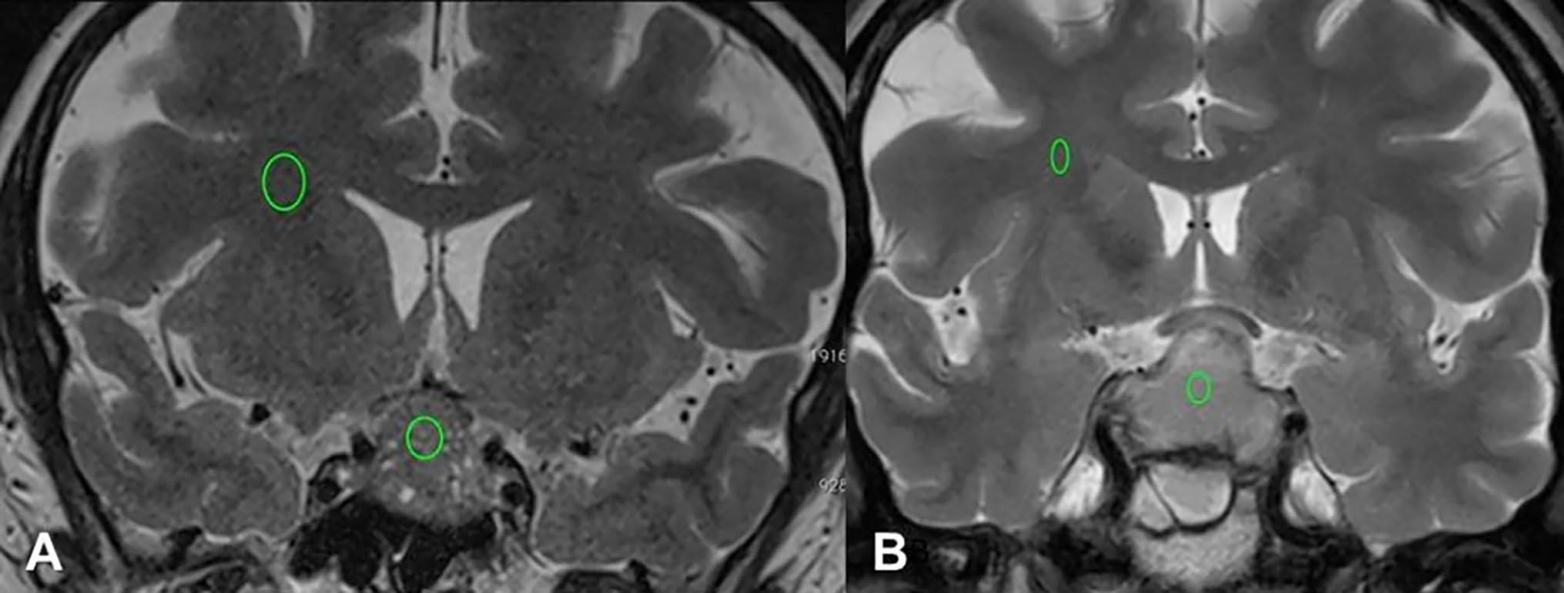
The role of T2 signal intensity-to-white matter ratio in corticotroph tumors. **A** A 52-year-old female with a diagnosis of densely granulated corticotroph tumor showed a pituitary tumor that occupied the sella and suprasellar cistern, with compression of the optic chiasm. The tumor appeared isointense to gray matter on T2-weighted imaging. The T2 signal-to-white matter ratio of the lesion was reduced to 0.026. **B** A 34-year-old female patient with a diagnosis of sparsely granulated corticotroph tumor was found to have a pituitary tumor on the sella and suprasellar cistern, with compression on the optic chiasm. The tumor appeared hyperintense to gray matter on T2-weighted imaging. The T2 signal-to-white matter ratio of the lesion was increased to 0.052

Thirty-six of 41 tumors could be visualized on MRI. Of the 5 patients whose tumors could not be visualized on MRI, 3 (60%) had DGCT and 2 (40%) had SGCT. The median (IQR) largest tumor size was 8.50 (9.75) mm in DGCTs compared to 16.00 (16.00) mm in SGCTs (*p* = 0.049). The median (IQR) tumor volume was 281.00 (898.25) mm^3^ in the DGCT group and 670.00 (3436.50) mm^3^ in the SGCT group. The tumor volume was bigger in SGCTs, but this did not yield statistical significance (*p* = 0.207). There was no significant difference in T2-WSI (hypointense/isointense/hyperintense) between tumor subtypes (*p* = 0.903). The mean T2 intensities (quantitative) were 667.33 ± 296.01 and 579.45 ± 410.31 in the DGCT and SGCT groups, respectively (*p* = 0.560). The mean white matter (quantitative) value was 561.83 ± 218.47 in the DGCT group and 359.09 ± 180.30 in the SGCT group (*p* = 0.025). The mean T2 intensity-to-white matter ratio was 0.03 ± 0.02 in the DGCT group and 0.05 ± 0.21 in the SGCT group, being significantly higher in the SGCT group (*p* = 0.049) (Fig. 2). There was no significant difference between DGCTs and SGCTs in terms of the Knosp or Hardy classification grades (invasive/non-invasive) or the Hardy classification stages (suprasellar/parasellar) (*p* > 0.005).

### Tumor Proliferation

Among the histopathological parameters evaluated in this study, the median (IQR) Ki-67 LI value was 2.00% (1.00%) in the DGCT group and 4.00% (7.00%) in the SGCT group (*p* = 0.043). The Ki-67 LI was ≥ 3% in 11 (57.9%) DGCTs and in 8 (42.1%) SGCTs (*p* = 0.184). The median (IQR) value of mitotic count per 2 mm^2^ was 0.00 (1.00) in the DGCT group and 1.00 (2.50) in the SGCT group (*p* = 0.001). The mitotic count was ≥ 2 per 2 mm^2^ in 1 (14.3%) DGCT and ≥ 2 per 2 mm^2^ in 6 SGCTs (85.7%). Tumors with mitotic counts of ≥ 2 per 2 mm^2^ were significantly more prevalent in the SGCT group (*p* = 0.002).

### Predictors of Early Biochemical Remission

A binary logistic regression model was created using variables identified as having significant differences between the groups in the univariate analysis (remission, tumor size, Ki-67 LI, and mitotic count). In the multivariate analysis, it was determined that the probability of early biochemical remission was lower in SGCTs independently of other significant parameters (OR 0.05, 95% CI 0.05–0.52, *p* = 0.012). Tumor size (OR 1.07, 95% CI 0.87–1.32, *p* = 0.506), Ki-67 LI (OR 0.97, 95% CI 0.58–1.63, *p* = 0.921), and mitotic count (OR 2.84, 95% CI 0.40–19.82, *p* = 0.291) were not predictive features of SGCTs (Table 2).

**Table 2:** Multivariate logistic regression analysis for prediction of sparsely granulated corticotroph tumors

	**OR (95% CI)**	***p*-value**
Remission	0.05 (0.05–0.52)	0.012^*^
Largest tumor size	1.07 (0.87–1.32)	0.506
Ki-67 labeling index	0.97 (0.58–1.63)	0.921
Mitotic count	2.84 (0.40–19.82)	0.291

Bold value denotes statistical significance

*CI* confidence interval, *OR* odds ratio

^*^Binary logistic regression analysis

## Discussion

This study provides additional support in the tumor granulation pattern of functional corticotroph tumors constituting an independent risk factor for early biochemical remission. SGCTs were shown to be larger and more proliferative tumors (based on both the Ki-67 LI and mitotic count) compared to DGCTs, and elevated T2 intensity-to-white matter ratio may serve as a preoperative radiological harbinger of SGCTs. These results emphasize the need for accurate histological subtyping of corticotroph tumors as indicated in the modern classification of pituitary neuroendocrine tumors [29, 31].

The importance of tumor granulation patterns has been long emphasized by disease experts in the field. Subsequent studies reported that, overall, DGCTs were more common, smaller, and less invasive tumors compared to SGCTs [3, 8–10]. Consistent with the literature, in our series, the majority of corticotroph tumors were DGCTs, and SGCTs were associated with larger tumor sizes. The current series also showed that both the Ki-67 LI and mitotic count were higher in SGCTs compared to DGCTs, which may explain why SGCTs are indeed larger and more invasive. In a former study, DGCTs were also reported to be more commonly associated with lower Ki-67 LI values (< 3%) and were characterized by lower Knosp grades; however, no mitotic counts were provided in that study [9]. In another study, higher tumor volumes were associated with higher Ki-67 LI and increased in SGCTs [10].

Some studies have demonstrated a correlation between elevated ACTH secretion and tumor volume in corticotroph tumors [10, 32]. This suggests that tumor subtypes may be more strongly related to preoperative ACTH levels than the tumor volume. In terms of tumor volume and ACTH levels, we observed higher tumor volumes and elevated ACTH levels in the SGCT group. However, this did not yield statistical significance, probably due to the relatively small number of patients in each group. Large series may provide additional insights into this matter.

Studies have reported remission rates after TSS ranging between 59 and 90% and recurrence rates between 3 and 46% [13, 16, 33, 34]. The variation in the data may be attributable to the use of different definitions of remission and recurrence, as well as varying durations of follow-up and sizes of the patient populations [21]. Furthermore, in studies predicting remission, the evaluation of different remission criteria such as postoperative immediate remission, early biochemical remission, or long-term remission after surgical therapy may also contribute to the variable rates reported by different researchers. In our study, the early biochemical remission rate was 63.4%, while the recurrence rate was 12.2%, and our findings were consistent with the literature. In the current series, the remission rate was higher in patients with DGCTs. There are several other studies in the literature showing that DGCTs are associated with postoperative remission. One of these studies was recently conducted on a large patient population, and the authors reported that patients with DGCTs achieved more frequently an immediate biochemical remission compared to patients with SGCTs [9]. Similarly, another series of 59 patients with CD reported DGCTs were associated with significantly higher rates of early biochemical remission compared to those of SGCTs [10]. Although the relationship between tumor granulation patterns and postsurgical remission remains unclear in somatotroph tumors [35], studies suggested that small tumor size increases the likelihood of achieving remission [36], a pattern that is likely applicable to corticotroph tumors. Moreover, our multivariate analysis showed that DGCTs were significantly associated with remission which was independent of variables such as tumor size and proliferative activity, likely due to the ease of resectability.

In terms of tumor size, corticotroph tumors are most often microtumors, the detection of which may be challenging in imaging studies since the tumors may be invisible using conventional MRI in as many as 36 to 64% of all cases. As a result, most studies on this subject have been designed to determine preoperative tumor localizations [37]. There is only one study examining the relationship between tumor subtypes and MRI findings. In that study, which evaluated 29 patients with CD, corticotroph tumors were generally seen as T2-hyperintense on MRI, and T2-hyperintense corticotroph tumors were associated with larger tumor sizes and sparsely granulated patterns [38]. However, tumors under 5 mm were not included in that study, and therefore, the number of DGCTs might have been underestimated. On the contrary, in our study, which had a larger patient population compared to the previous study, T2-hyperintense tumors were in the minority, and there was no relation between tumor subtype and T2-WSI. However, the T2-WSI assessment (hypointense/isointense/hyperintense) has a noteworthy margin of error as it is dependent on the radiologist and imaging tools. It has also been shown in the literature that combinations of MRI parameters can provide more accurate diagnostic information in the differential diagnosis of some diseases [23]. In order to perform a more objective evaluation, we also compared quantitative T2 intensity values, quantitative white matter values, and quantitative T2 intensity-to-quantitative white matter ratios between DGCTs and SGCTs. Although we did not obtain significant results in terms of quantitative values of T2 intensity between tumor groups, interestingly, we found that quantitative white matter values were significantly lower, and T2 intensity-to-white matter ratios were significantly higher in cases of SGCTs compared to DGCTs. This finding is of clinical significance considering that sparse tumor granulation patterns of corticotroph tumors can only be diagnosed during the histopathological examination, and there are no preoperative biochemical markers to distinguish these tumors. The relationship between the sparsely granulated pattern in somatotroph tumors and the demonstration of T2-hyperintensity on preoperative MRI raises questions on whether a similar relationship may also exist for corticotroph tumors [38]. The demonstration of such a relationship in future studies may contribute to preoperative disease management. However, a higher quantitative T2 intensity-to-white matter ratio of SGCTs seems to be a promising radiological harbinger identified in our series.

Because functional corticotroph tumors are often microtumors on MRI studies, pituitary apoplexy is an uncommon manifestation. A recent retrospective study confirmed that apoplexy in CD is more commonly associated with macrotumors [39]. In our study, pituitary apoplexy developed before pituitary surgery in two affected patients in the group of DGCT. These measure 17 mm and 22 mm. This is an interesting finding because DGCTs most often tend to be microtumors. In contrast to our study, a recently published case report on the SGCT subtype described postoperative remission after apoplexy [40]. Since the number of case reports in the literature addressing the development of apoplexy in CD is still limited, the relationship between apoplexy and corticotroph tumor subtypes remains unclear.

The limitations of our study stem from its retrospective nature and small number of patients. In addition, our study did not have a long follow-up period to evaluate long-term remission and recurrences. However, our study is still valuable in addressing multidisciplinary clinicopathological correlates of DGCTs and SGCTs given the paucity of well-documented series.

## Conclusion

Our data support the rationale of detailed tumor subtyping based on granulation patterns of functional corticotroph tumors since the tumor granulation patterns correlate with tumor size, tumor proliferative activity, and early biochemical remission. Moreover, our study identified that elevated T2 intensity-to-white matter ratio may serve as a radiological harbinger of SGCTs.

## Data Availability

The data analyzed for this study are not publicly available according to relevant regulations but may be obtained from the corresponding author upon reasonable request.
